# Contrasting Responses of Rhizosphere Fungi of *Scutellaria tsinyunensis*, an Endangered Plant in Southwestern China

**DOI:** 10.1128/spectrum.00225-22

**Published:** 2022-07-05

**Authors:** You-wei Zuo, Feng-qiong Yu, Jia-hui Zhang, Chang-ying Xia, Huan Zhang, Hong-ping Deng

**Affiliations:** a Center for Biodiversity Conservation and Utilization, School of Life Sciences, Southwest University, Beibei, Chongqing, China; b Chongqing Key Laboratory of Plant Resource Conservation and Germplasm Innovation, Institute of Resources Botany, School of Life Sciences, Southwest University, Beibei, Chongqing, China; c Chongqing Academy of Science and Technology, Low Carbon and Ecological Environment Protection Research Center, Chongqing, China; University of Minnesota

**Keywords:** *Scutellaria tsinyunensis*, altitude, rhizosphere, fungal communities, soil properties

## Abstract

*Scutellaria tsinyunensis* is an endangered species in southwest China, distributed sporadically in mountainous areas at an elevation of approximately 200 to 900 m. Rhizosphere soil properties and fungal communities play critical roles in plant survival and expansion. Nevertheless, understanding of soil properties and fungal communities in the *S. tsinyunensis* distribution areas is extremely limited. The present study examined soil properties and fungal communities in nearly all extant *S. tsinyunensis* populations at two altitudinal gradients (low and high groups). Our findings indicated that soil characteristics (i.e., soil pH, water content, and available phosphorus) were affected distinctively by altitudes (*P < *0.05). In addition, the low altitude group harbored higher fungal richness and diversity than the high altitude. Co-occurrence network analysis identified six key genera that proved densely connected interactions with many genera. Further analysis represented that the low altitude group harbored three beneficial genera belonging to Ascomycota (*Archaeorhizomyces*, *Dactylella*, and *Helotiales*), whereas the high altitude showed more pathogenic fungi (*Apiosporaceae*, *Colletotrichum*, and Fusarium). Correlation analysis found that soil water content was highly correlated with *Hydnodontaceae* and *Lophiostoma*. Besides, plants’ canopy density was negatively correlated with four pathogenic fungi, indicating that the high abundance of the pathogen at high altitudes probably inhibited the survival of *S. tsinyunensis*. To sum up, this comprehensive analysis generates novel insights to explore the contrasting responses of *S. tsinyunensis* rhizosphere fungal communities and provides profound references for *S. tsinyunensis* habitat restoration and species conservation.

**IMPORTANCE** Our study highlighted the importance of rhizosphere fungal communities in an endangered plant, *S. tsinyunensis*. Comparative analysis of soil samples in nearly all extant *S. tsinyunensis* populations identified that soil properties, especially soil water content, might play essential roles in the survival and expansion of *S. tsinyunensis*. Our findings proved that a series of fungal communities (e.g., *Archaeorhizomyces*, *Dactylella*, and *Helotiales*) could be essential indicators for *S. tsinyunensis* habitat restoration and protection for the first time. In addition, further functional and correlation analyses revealed that pathogenic fungi might limit the plant expansion into high altitudes. Collectively, our findings displayed a holistic picture of the rhizosphere microbiome and environmental factors associated with *S. tsinyunensis*.

## INTRODUCTION

*Scutellaria* includes at least 300 species, and most of them are distributed globally except for tropical Africa (1). Previous studies have demonstrated that *Scutellaria* can be used in Traditional Chinese Medicine to cure liver and lung complaints (2, 3). The primary components of *Scutellaria* include a series of flavonoids, such as wogonin and baicalin, harboring antioxidative, antiphlogistic, and hemostatic effects (4). *S. tsinyunensis* (Lamiaceae), as a valuable endemic plant, distributes only in the Jinyun Mountain, Chongqing, China. Currently, the distribution area of *S. tsinyunensis* is restricted to 1.5 km^2^ and ranges from approximately 500 to 800 m altitudes (5). Nonetheless, *Scutellaria*, particularly *S. tsinyunensis*, has been growingly threatened by human activities such as habitat transformation and harvesting. It is well-documented that wild plants are a critical strategic resource for the current society, whereas the extinction of a wild variant can reduce the genetic resources of the species and induce a chain reaction in its survival network (6). Hence, it is essential to provide new insights into the endangered plant and promote its population prosperity comprehensively.

Soil abiotic factors are the most important drivers of plant growth, survival, and nutrient cycling (7, 8). Specifically, soil pH, organic matter (OM), water content (WC), available nitrogen (AN), and phosphorus (AP) are the dominant soil variables. Among these, soil WC, for instance, is the distinctive contributor to plant distribution and plays a critical role in promoting nutrient cycling, keeping environmental moisture, and strengthening soil ventilation (9). As a microenvironment of plant roots, the plant’s rhizosphere is an effective site for soil-root-microorganism interaction (10). It harbors a variety of microorganisms (e.g., soil bacteria, fungi, and archaea), which in turn induce the metabolism of nutrients and increase the livability of plants (11). Therefore, investigating soil properties and rhizosphere microbiome provides profound insights into the living conditions of *S. tsinyunensis*.

Rhizosphere microorganisms include many beneficial microorganisms, which can play a profound role in plant growth and development (11). It is worth noting that fungi can form symbionts with plant roots, thus, promoting the growth and development of plants, increasing rhizosphere organism metabolism, and helping to reduce the use of pesticide fertilizer (12, 13). In addition, plant roots can create unique soil microhabitats by changing pH and oxygen concentrations in the soil and promoting the prosperity of rhizosphere microbial taxa (14). Growing evidence has shown an efficient protective strategy to build purposeful conservation of fungal communities in places where endangered plants exist. For instance, the protection of the fungus *Rhizopogon yakushimensis* can simultaneously protect a highly endangered species, *Pinus amamiana* (15). In addition, some fungi, such as *Trichoderma*, *Mortierella*, and *Hypocrea*, were assumed to protect the first-class endangered plant *Cypripedium japonicum* and enhance its livability (16). Therefore, the investigation of rhizosphere fungal communities can provide essential understanding for the survival and protection of rare and endangered species.

Thus, to investigate the relationship between the rhizosphere fungal communities and *S. tsinyunensis*, we yielded rhizosphere soil samples from eight populations of *S. tsinyunensis* divided by two altitudinal levels. Here, we hypothesized that fungal composition and diversity would alter due to spatial variations and that distinctive fungal communities would respond significantly to soil and plant properties. Specifically, the primary focuses of this study aim to (i) uncover how soil properties respond to altitudinal variation; (ii) identify the key soil fungal community and explore how altitude reshapes fungal composition and function; and (iii) evaluate the relationships between fungal communities and soil and plant properties. To sum up, this study provides essential references for understanding soil properties and fungal communities in *S. tsinyunensis* and offers valuable resources for habitat restoration and species conservation.

## RESULTS

### Soil properties were altered between two altitudes.

**FIG 1:**
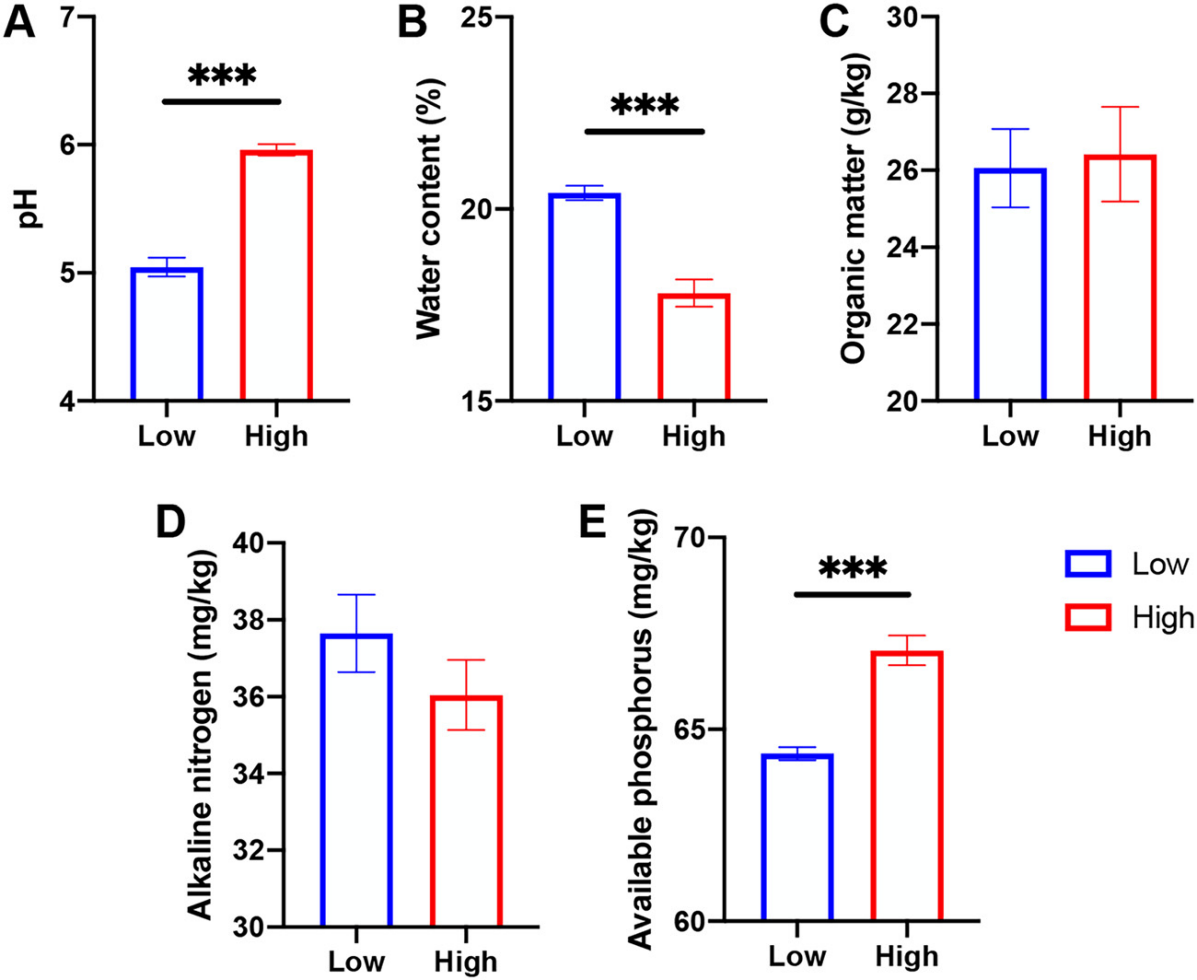
Soil properties were altered between two altitudes. Alterations of (A) soil pH, (B) water content, (C) organic matter, (D) available nitrogen, and (E) available phosphorus under low and high altitudinal groups. *** Asterisk indicates the significant difference (*P < *0.001).

Soil characteristics were affected distinctively by altitudes but showed different trends (Fig. 1). Specifically, soil pH and AP were higher at high altitude (800 m) than at low altitude (600 m) (*P < *0.001), whereas soil WC was significantly decreased at high altitude (*P < *0.001). Nonetheless, the other two soil properties (i.e., soil OM and AN) showed no significant alterations between the comparison (*P > *0.05).

### Composition and diversity of rhizosphere fungal community in *S. tsinyunensis*.

**FIG 2:**
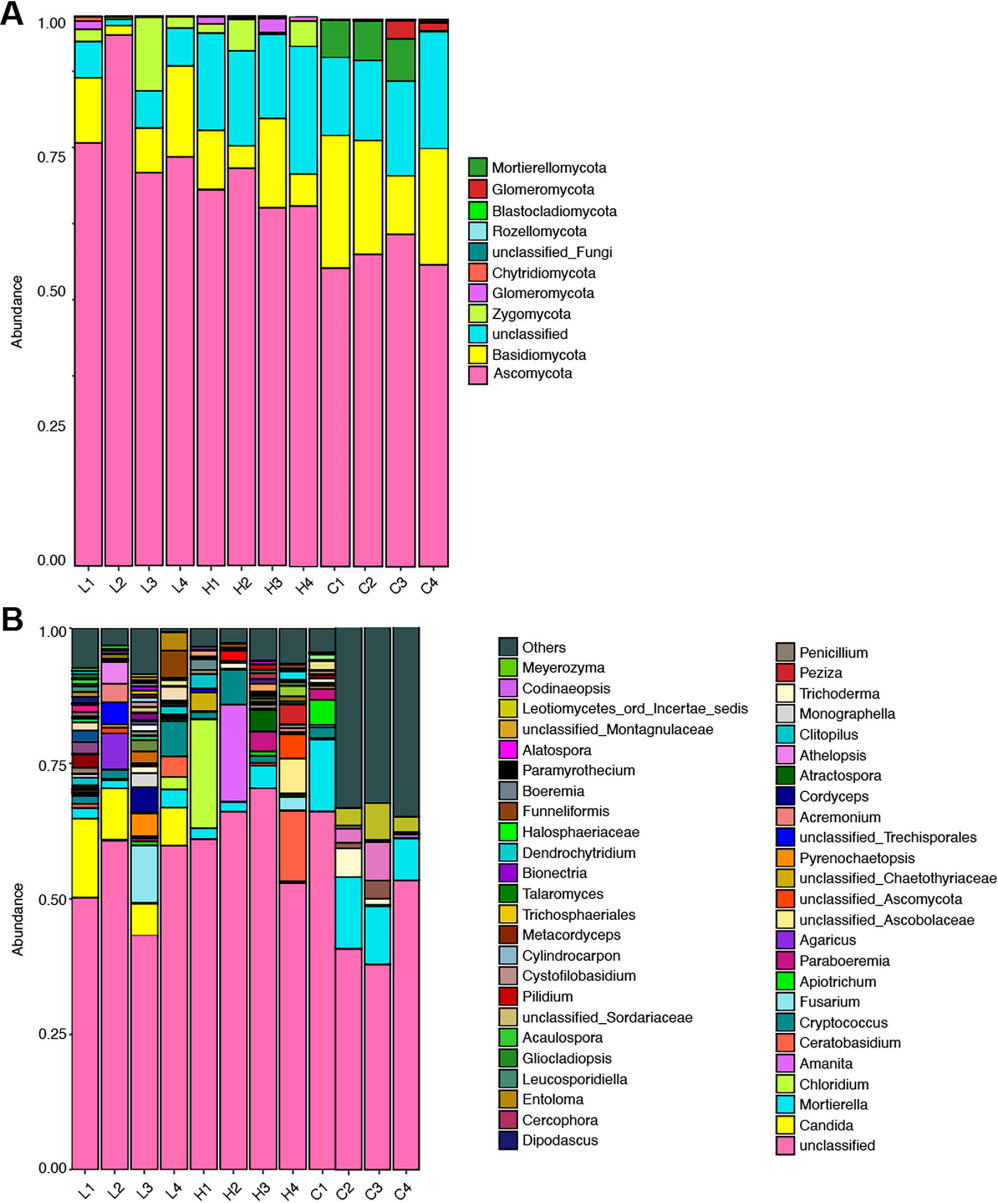
Taxonomic distribution of fungal communities. Bar plots show the fungal community composition at phylum (A) and genus (B) levels. The order of fungal communities was listed in decreasing order based on the sum of their relative abundance in all replicates. L1 to L4, low altitude; H1 to H4, high altitude; C1 to C4, control group.

A total of 636,173 effective sequences (with an average of 53,014 sequences) and 567,230 optimizing sequences (with an average of 47,269 sequences) were yielded from all soil samples. In addition, the OTU number of samples ranged from 265 to 717, with an average of 500 ± 176 (mean ± SD). For the rhizosphere fungal composition of *S. tsinyunensis*, at the phylum level, the dominant fungal phyla were Ascomycota and Basidiomycota (Fig. 2A). Compared with the control group, Ascomycota in rhizosphere soils of *S. tsinyunensis* were increased, whereas Basidiomycota decreased (*P < *0.05). At the genus level, the dominant fungal communities were *Candida* and *Mortierella* (Fig. 2B). Among them, the relative abundance of *Candida* distributed in low altitude was extremely higher than that of high altitude and control groups (*P < *0.05). On the contrary, Fusarium showed higher relative abundances at both high altitude and control groups than at low altitude (*P < *0.05).

**FIG 3:**
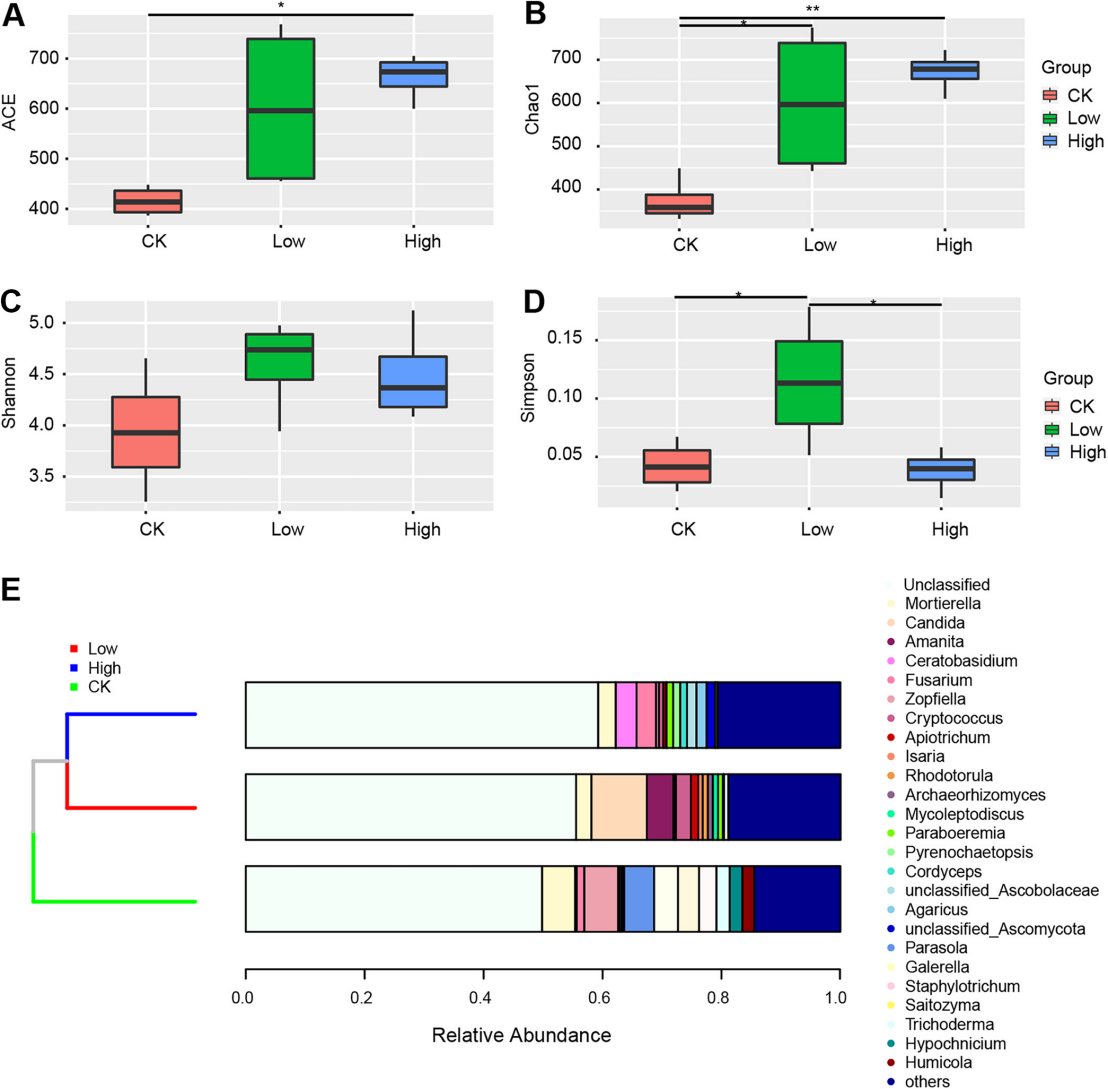
Diversity indexes of various groups of rhizosphere fungal communities. General patterns of fungal alpha-diversity with altitudinal gradients, according to ACE (A), Chao1 (B), Shannon (C), and Simpson (D) indexes. */** Asterisks indicate the significant difference (*P < *0.05/0.01). (E) Unifrac tree showing the distances of the identified fungal genera.

ACE, Chao1, Shannon, and Simpson indexes were used to evaluate the community richness and diversity of *S. tsinyunensis* rhizosphere fungi. The results showed that the fungal richness and diversity were elevated in two rhizosphere groups compared with the control group. Specifically, ACE and Chao1 indexes were higher in the low and high altitudinal groups (Fig. 3A, B). Interestingly, the Shannon index was higher in both two rhizosphere groups but showed no significant differences, whereas the Simpson index was highest in the low altitude group compared with the control group and high altitude group (Fig. 3C, D). The present study subsequently analyzed the beta diversity to evaluate further the similarity and difference of variations in the soil fungal community. Unweighted pair-group method with arithmetic means (UPGMA) clustering trees based on taxonomy presented that two rhizosphere groups were highly clustered at genus levels (Fig. 3E), whereas the control group showed a distinctive branch of the clustering tree.

### Co-occurrence analysis of rhizosphere fungal community in *S. tsinyunensis*.

**FIG 4:**
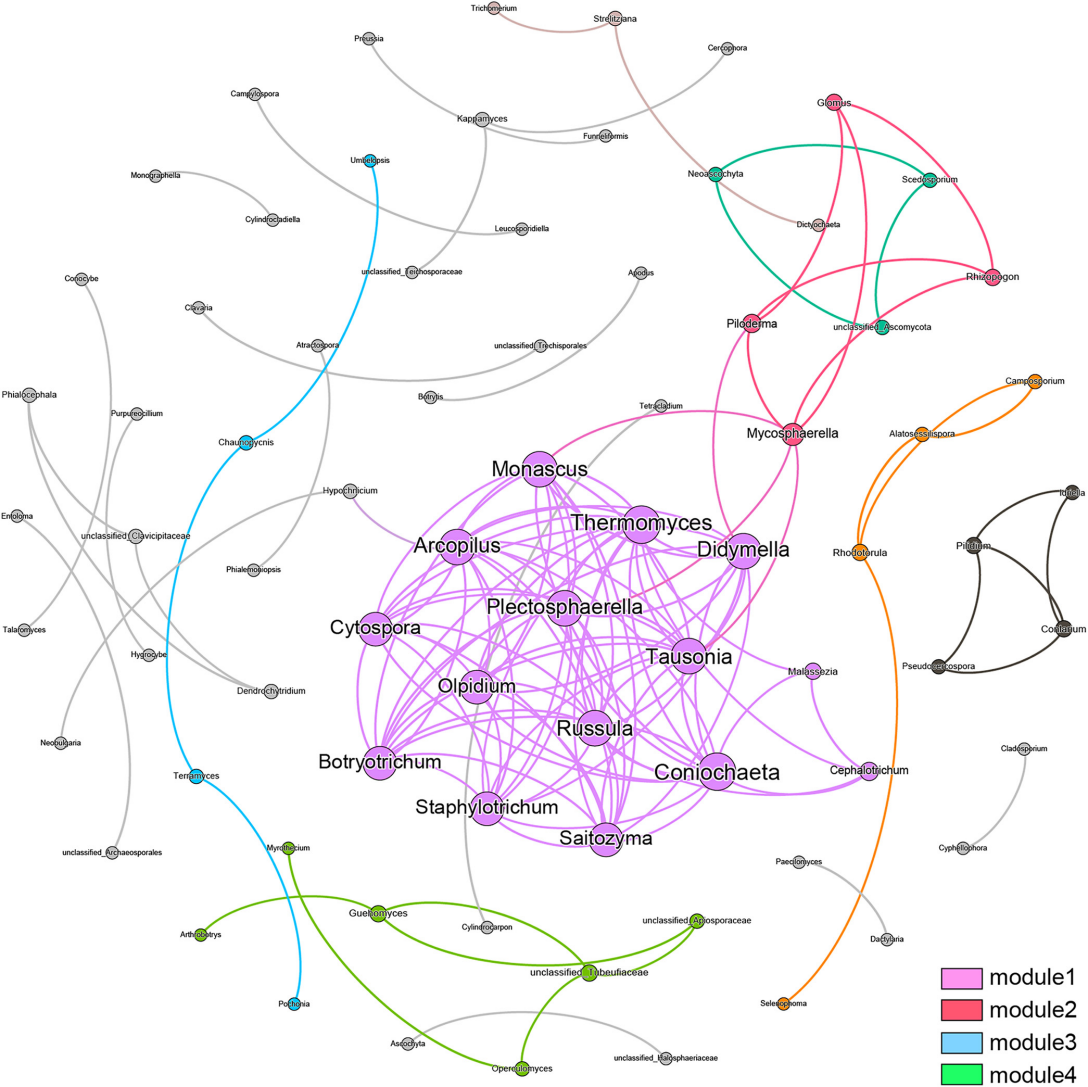
Co-occurrence analysis of fungal community. The colors of the nodes indicate modularity class, and most genera belong to modules 1 to 4. The size of nodes indicates the relative abundance of fungal taxa. Each connection represents a strong correlation (Spearman correlation coefficient > 0.75 and *p-adj *< 0.01).

The present study constructed network analysis to investigate the co-occurrence patterns in the soil fungal community (Fig. 4). The network consisted of 77 nodes and 137 edges. The modularity index was 0.554, indicating a modularization in the generated network. In addition, the network diameter was six edges, with an average path length of 1.75. The whole network could be divided into four distinctive modules after modularized distributions. Notably, module1 harbored the most connected OTUs (15) and the highest density. A total of 10 nodes in module1 belonged to Ascomycota, four nodes belonged to Basidiomycota, and one node belonged to Olpidiomycota. The top six genera, *Coniochaeta* (1), *Thermomyces* (1), *Monascus* (0.986), *Plectosphaerella* (0.986), *Tausonia* (0.986), and *Russula* (0.985), were identified as hub nodes due to their high eigenvector centrality scores.

### Significant difference of rhizosphere fungal composition and function in *S. tsinyunensis*.

**FIG 5:**
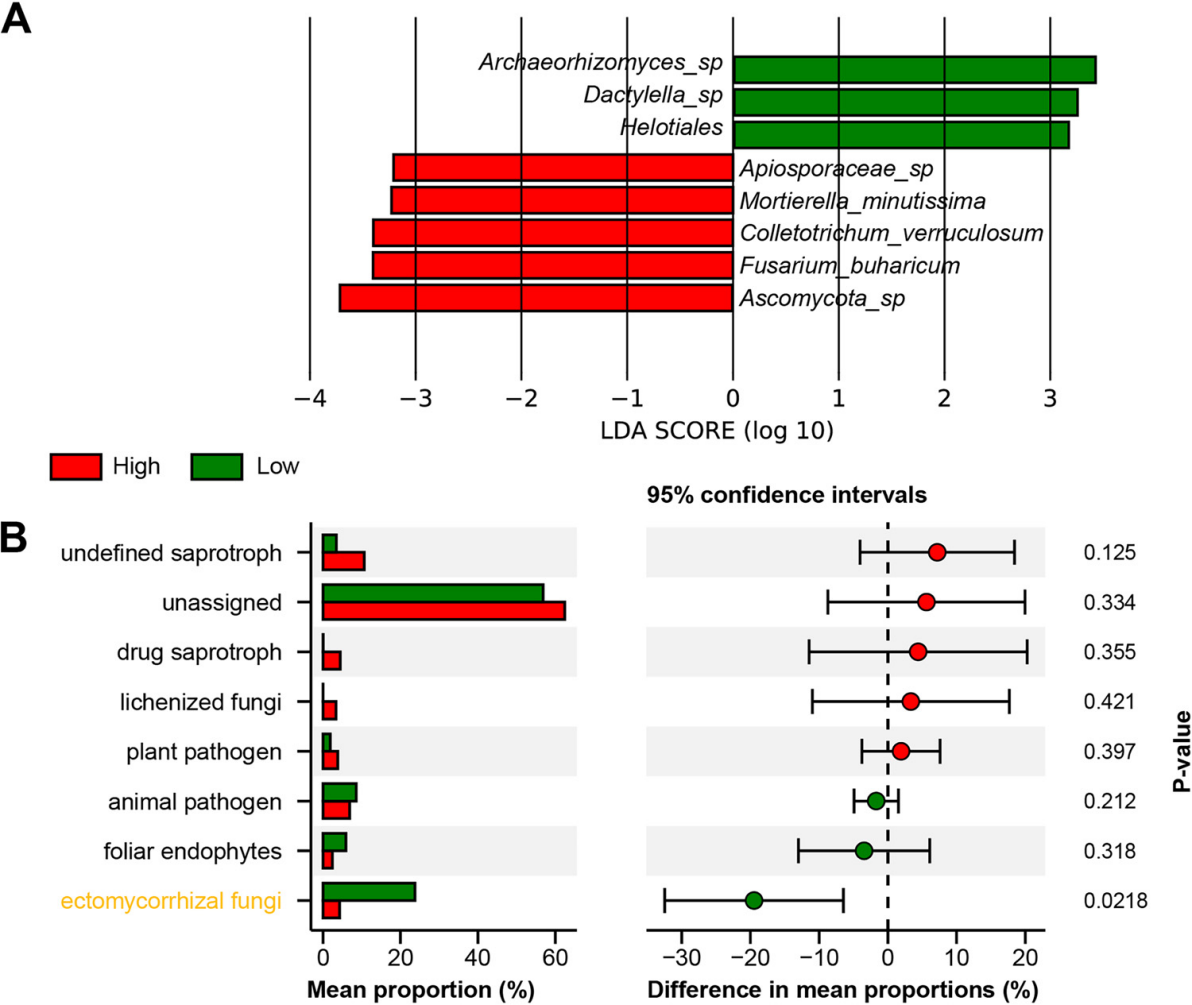
The composition and function of rhizosphere fungal communities were diverse at different altitudinal gradients. (A) LDA analysis showed specific differences in rhizosphere fungal taxa. (B) Guild assignments for the yielded OTUs using FUNGuild. The mean proportion of fungal function was calculated to indicate relative abundance in two altitudinal groups.

LDA analysis was performed to uncover specific differences in rhizosphere fungal taxa between two altitudinal groups (Fig. 5A). According to the results, three taxa belonging to Ascomycota (*Archaeorhizomyces_sp*, *Dactylella_sp*, and *Helotiales*) showed distinctively higher abundance in the low altitude group. Nonetheless, the other four taxa belonging to Ascomycota (Fusarium*_buharicum*, *Ascomycota_sp*, *Colletotrichum_verruculosum*, and *Apiosporaceae_sp*) and one taxon belonging to Zygomycota (i.e., *Mortierella_minutissima*) displayed significantly higher abundance in high altitude group. In addition, FUNGuild analysis was conducted to explore specific differences in rhizosphere fungal functions between two altitudinal groups (Fig. 5B). Functional analysis showed that 43.17% (low altitude) and 37.55% (high altitude) OTUs could be identified, indicating a large number of unclassified fungi in the rhizosphere soil of *S. tsinyunensis*. Notably, we found that ectomycorrhizal fungi showed higher relative abundance in the low altitude group (23.73%) than in the high altitude group (4.26%). The other functional fungi, such as undefined saprotroph, drug saprotroph, lichenized fungi, and plant pathogen, showed higher relative abundances in the high altitude group, whereas animal pathogen and foliar endophytes showed higher relative abundances in the low altitude group.

### Relationships of rhizosphere fungi with soil properties and plant population attribute.

**FIG 6:**
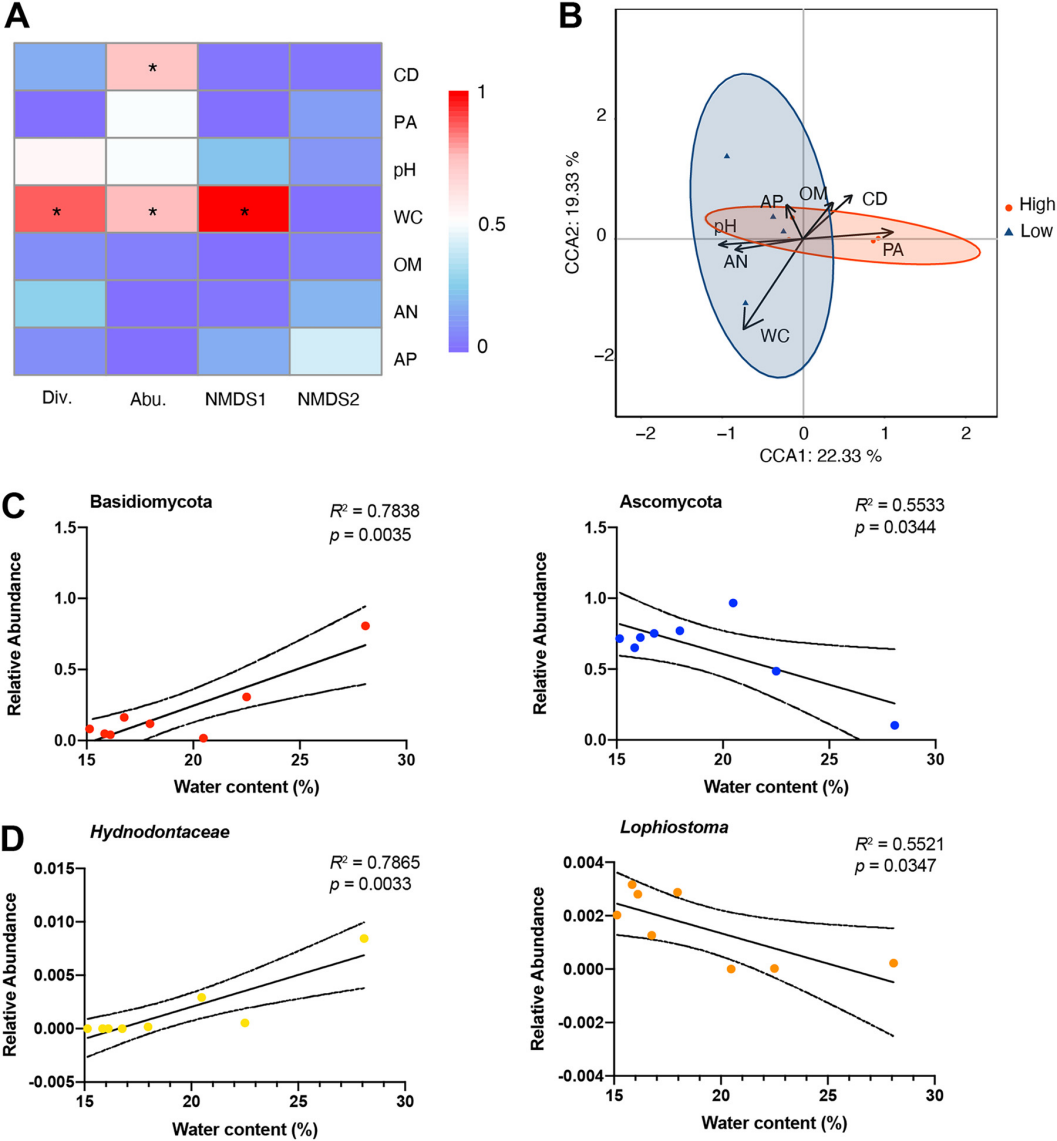
The relationships of fungal taxa with soil properties. (A) Heatmap showed the relationships of fungal diversity indexes with soil properties based on the Spearman rank correlation analysis. (B) The canonical correlation analysis showed the tendency of variables on different altitude levels. (C, D) Linear regression analysis identified specific fungal phyla (C) and genera (D) explained by soil water content.

Spearman correlation analysis was conducted on the soil and plant’s properties and rhizosphere fungal alpha and beta diversities (Fig. 6A). The results showed that soil WC was significantly correlated with fungal richness and diversity (*P < *0.05), and plants’ canopy density was distinctively correlated with fungal richness. Besides, CCA analysis further validated that soil WC dramatically explained the fungal alterations from low altitude, whereas canopy density could explain the alterations from high altitude (Fig. 6B). All soil and plant properties could explain a total of 41.66% fungal differences.

**FIG 7:**
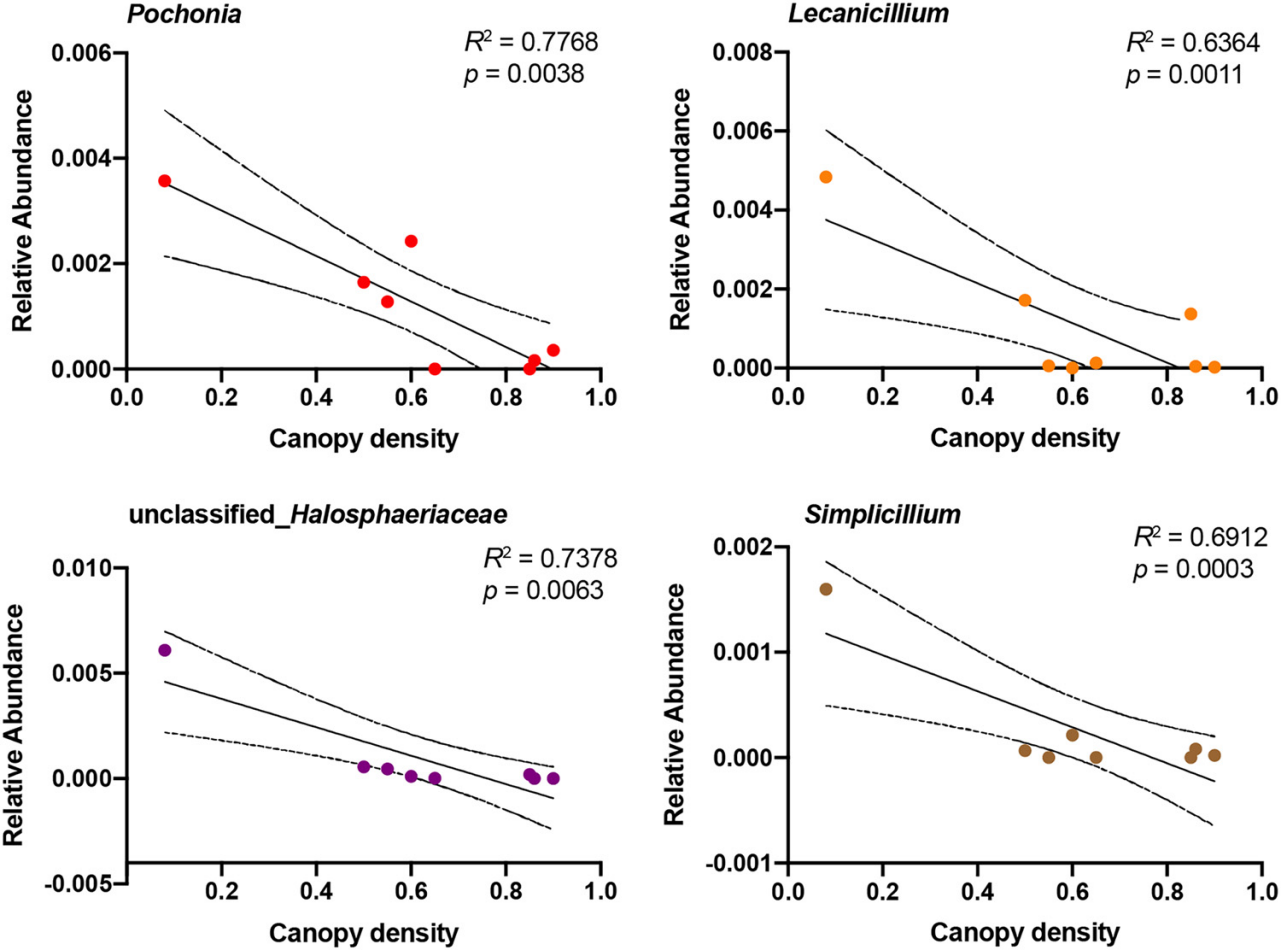
Linear regression analysis identified specific and genera explained by canopy density.

We then performed regression analysis to identify the specific fungal phyla and genera explained by soil WC and plant’s canopy density. The results showed that soil WC was dramatically contributed to Basidiomycota (positive correlation, *R*^2^ = 0.7838, *P = *0.0035) and Ascomycota (negative correlation, *R*^2^ = 0.5533, *P = *0.0344) (Fig. 6C). At the genus level, soil WC was primarily contributed to *Hydnodontaceae* (positive correlation, *R*^2^ = 0.7865, *P = *0.0033) and *Lophiostoma* (negative correlation, *R*^2^ = 0.5521, *P = *0.0347) (Fig. 6D). Interestingly, no fungal phylum could be explained by plants’ canopy density, except four genera, *Pochonia* (*R*^2^ = 0.7768, *P = *0.0038), *Lecanicillium* (*R*^2^ = 0.6364, *P = *0.0011), unclassfied_*Halosphaeriaceae* (*R*^2^ = 0.7378, *P = *0.0063), and *Simplicillium* (*R*^2^ = 0.6912, *P = *0.0003), were highly negative correlated with the variant (Fig. 7).

## DISCUSSION

Soil microorganism is an indispensable biological factor in soil ecological processes, such as soil nutrient transformation, leaf, and root decomposition, and plant growth and survival (17, 18). The community composition and diversity of rhizosphere microorganisms directly affect plants’ absorption of water and nutrients (19). Hence, the primary objective of this research was to estimate the composition and diversity of the rhizosphere fungal community and demonstrate the relationships between specific soil and plant properties with the identified fungi.

### Soil properties were altered at different altitude levels.

In this study, we identified that the soil properties (i.e., pH, OM, and AP) showed distinctive differences at different altitudes, whereas soil OM and AN represented no alterations between the two altitudinal groups. Soil is a dynamic system with a large number of materials, and soil fertility is the indicator of the plant’s productivity (20). For example, soil pH, known as one of the most dominant predictors of rhizosphere microbial community, can be driven by transportation and transformation in the form of N (21, 22). As a result, the process promotes the livability and harvesting of plants and crops (23). Nonetheless, previous evidence has shown that the suitable soil pH for *S. tsinyunensis* was 3.96 to 5.36 (24). Hence, the relatively high pH value identified at high altitude might restrict the expansion of *S. tsinyunensis.* Importantly, our results showed that soil WC was depleted in the high altitude group compared with the low altitude group. It was reported that sufficient WC could induce Moso bamboo easily invading into the original habitat of *Alsophila spinulosa*, and relatively low WC vitiated the expansion and survival of *Scutellaria* species (25, 26). Our results were also consistent with a previous report that demonstrated a decrease in soil WC resulted in a reduction of new ramet formation, a shorter tree height, and decreased total biomass (27). Therefore, lower WC content at high altitudes might be the dominant factor limiting the population expansion of *S. tsinyunensis*.

### Composition and diversity of rhizosphere fungal community were diverse at different altitude levels.

Soil microorganisms play an essential role in regulating soil fertility, affecting the growth and development of plants, and participating in the transformation of soil organic matter, nutrient cycling, and degradation of toxins secreted by roots. In order to further explore the relationship between rhizosphere microorganisms (especially fungi) and soil and plant properties, we analyzed the rhizosphere fungal community characteristics of *S. tsinyunensis* at different altitudes. Our results showed that Ascomycota and Basidiomycota were the dominant phyla identified in the two rhizosphere groups. Fungi from the Ascomycota are effective contributors in controlling plant pathogens, promoting plant development, and regulating carbon and nitrogen cycling in arid ecosystems (28, 29). Similarly, fungi from Basidiomycota are well documented as promising agents for carbon recycling, ecosystem functioning, and medicinal properties (30). In addition, the co-occurrence network displayed that the most connected module (module 1) harbored 66.7% OTUs involved in Ascomycota. Based on the eigenvector centrality scores, we identified six nodes (i.e., *Coniochaeta*, *Thermomyces*, *Monascus*, *Plectosphaerella*, *Tausonia*, and *Russula*) as the hub genera in the study, indicating that these genera might contribute significantly to the composition and diversity of fungal communities. The genus *Coniochaeta*, *Plectosphaerella*, and *Tausonia* are known as woody hosts and soilborne pathogens (31–33). *Thermomyce* plays an important role in hemicellulose degradation and is a representative lignocellulose degrader (34). The genus *Monascus* has essential economic and ecological values, and has received wide attention owing to its multiple by-products and beneficial metabolites for food fermentations (35). Species of the *Russula* are important ectomycorrhizal fungi, which play a key role in soil nutrient uptake and cycling. Hence, these six taxa might have critical functions in reshaping the fungal community in the rhizosphere.

This study found that altitudinal levels exerted a significant effect on the fungal community diversity of *S. tsinyunensis* rhizospheric soil. Based on the alpha diversity indexes, the study identified that the fungal richness and diversity were elevated in two rhizosphere groups compared with the control group. In addition, the low altitude group harbored higher richness and diversity compared with the high altitude group. Our results were in line with a previous study that reported the abundance and phylogenetic diversity of microbial communities in the Rocky Mountains of Colorado decrease with the altitude (36). Similarly, alpine and subalpine altitude soil microbial diversity decreased with increasing altitude (37). Hence, it was speculated that the abundance and diversity of fungal communities were critical for the survival of *S. tsinyunensis*.

### Differences of rhizosphere fungal taxa and function in different altitude levels.

Due to geographic separation, soil microbial communities tend to show population stratification, such as altitudinal gradients, vertical depth, and horizontal distances. Among them, altitudinal gradients distinctively alter soils' microbial composition and diversity by affecting plant and soil properties compared with fine-scale spatial variations. In the present study, the LDA results showed that three beneficial taxa belonging to Ascomycota (*Archaeorhizomyces*, *Dactylella*, and *Helotiales*) were only abundant in the low altitude group. *Archaeorhizomyces* and *Dactylella* are widespread fungal taxa in many soil environments and can elevate plant bioactive compositions, promote stress tolerance, and alleviate the occurrence of plant diseases (38, 39). The genus of *Helotiales* comprises a large number of ectomycorrhizal fungi and plays a pivotal role in providing soil N/P to their hosts (40, 41). Interestingly, the high altitude group harbored three pathogenic taxa that could vitiate the survival and growth of *S. tsinyunensis*. Specifically, *Apiosporaceae* and *Colletotrichum* are endophytes, saprobes, and important plant pathogens, and have been proved to participate in plant diseases, such as soybean anthracnose (42, 43). Besides, pathological results illustrated that Fusarium sp. could trigger vascular wilt and root and crown rot symptoms on *Hibiscus moscheutos* (44). Hence, it was reasonable to assume that the low altitude group harbored more beneficial fungi. In contrast, the high altitude group showed more pathogenic fungi, and the differences in functional fungi might partially explain why the distribution of *S. tsinyunensis* decreases with elevation. The FUNGuild analysis supported our view that the low altitude group displayed a higher relative abundance of ectomycorrhizal fungi, promoting nutrient cycling and inducing systemic resistance against pests on host plants (45, 46).

### Relationships of fungal communities with soil properties and population attributes of *S. tsinyunensis*.

The soil physicochemical results showed that the soil WC was decreased with altitude, which would inevitably alter the abundance and diversity of rhizosphere fungal communities. According to the linear regression analysis, the abundance of *Hydnodontaceae* (Basidiomycota) was positively correlated with soil WC, whereas the abundance of *Lophiostoma* (Ascomycota) was negatively correlated with the variation. This finding was in agreement with a previous report that demonstrated *Hydnodontaceae* was negatively correlated with the death rate of *Panax notoginseng*, which can be considered an antagonist of pathogens (47). Intriguingly, strains of the *Lophiostoma* have been reported as endophytic fungi in a variety of plants, and metabolites generated from the fungi were reported to display phytotoxic effects and activity against various microorganisms (48, 49). Accumulating evidence has demonstrated that soil WC reduces microbial activity by altering diffusion of soluble substrates, microbial movement, and intracellular water potential. Hence, soil WC might determine *Hydnodontaceae* and *Lophiostoma* communities by managing nutrient availability and cell movement, consistent with a previous study that reported that soil WC connects soil particles might affect soil microbial structure patterns (50).

Canopy density is an essential indicator of the degree of canopy closure and space utilization. Previous studies have shown that the canopy density of *S. tsinyunensis* was decreased with altitude, and the value at around 800 m (0.545) was distinctively lower than that of low altitude (0.703) (24). The present study identified that rhizosphere fungal abundance was correlated with canopy density, and three classified genera (*Pochonia*, *Lecanicillium*, and *Simplicillium*) were negatively correlated with plant’s canopy density. Expectantly, these fungal communities are well-documented as pathogenic fungi or plant parasitic fungi, which can induce plant defense mechanisms and local resistance in fungal-nematode-plant interactions (51–53). Therefore, the high abundance of the pathogen at high altitude probably vitiates the defense mechanisms of plants, disrupts plant nutrient cycles, and causes damage to plant tissues.

To further uncover the role of soil fungal communities in the survival and expansion of *S. tsinyunensis*, it would be necessary to contrast its rhizosphere soil fungal community in more native and nonnative habitats in future research. To better understand the abiotic and biotic responses of the rhizosphere of *S. tsinyunensis*, investigations of the diversity and richness of other microorganisms (e.g., denitrifying bacteria, arbuscular mycorrhizal fungi, ectomycorrhizal fungi, and azotobacteria) in rhizosphere soil should include in our coming studies.

### Conclusion.

The present study investigated the rhizosphere fungal communities of nearly all existing populations of *S. tsinyunensis* and found that altitude and soil WC were essential drivers affecting the fungal community structures. Our results indicated that the abundance of fungi genera at different altitudes was distinct: low altitude harbored more beneficial fungi, whereas high altitude harbored more pathogenic fungi, which might partially explain why the distribution of *S. tsinyunensis* decreases with elevation. In addition, our findings identified a series of fungal communities (e.g., *Archaeorhizomyces*, *Dactylella*, and *Helotiales*) for evaluating the survival and expansion of *S. tsinyunensis*. Collectively, these results highlight the importance of rhizosphere fungal communities of *S. tsinyunensis*, and provide profound insights into the altitudinal pattern and soil drivers of fungal communities in ecosystems.

## MATERIALS AND METHODS

### Community survey and soil samples collection.

Jinyun Mountain National Nature Reserve is located in Beibei District, Chongqing, China (106°22′18″ to 106°24′42″E, 29°45′25″ to 29°51′53″N). The protected area belongs to a midsubtropical humid monsoon climate, with 13.6°C annual average temperature, 805.9 mm annual average precipitation, and 87% relative humidity. There are at least 1,774 species of vascular plants belonging to 890 genera and 204 families in the protected area, characterized by *S. tsinyunensis*, *Begonia jinyunensis*, and *Euonymus chloranthoides*.

Considering the rarity of *S. tsinyunensis*, four sites (L1 to L4) at lower elevations (around 600 m) and four sites (H1 to H4) at higher elevations (around 800 m) were identified in the protected area, respectively. The *S. tsinyunensis* population at around 600 m was 916, whereas the number reduced to 307 at around 800 m. Soil samples from four sites distant from *S. tsinyunensis* populations were labeled as control samples (CK1 to CK4). In order to investigate the composition and function of the rhizosphere fungi, eight rhizosphere soil samples of *S. tsinyunensis* from low (600 m) and high (800 m) sites were excavated and shaken carefully to separate the soil from the roots. The collected samples were immediately housed in an insulated container with ice and then transported to the laboratory. The soil samples were well ground and sieved (<2 mm) after removing debris and roots. A portion of the soil samples was stored at −80°C for subsequent sequencing analysis, whereas the remaining soil was used for soil physical and chemical characterization. In addition, canopy density and population area for each sampling site were recorded accordingly.

### Soil physical and chemical properties.

Physical and chemical analyses for soil pH, OM, WC, alkaline nitrogen (AN), and available phosphorus (AP) were conducted as previously reported (54). Specifically, after standardized treatment, soil WC was determined by drying method; soil pH was determined by potentiometric method; soil OM was measured by potassium dichromate and sulfuric acid digestion method; soil AN was measured by alkali hydrolysis diffusion method; and soil AP was quantified by NaHCO_3_ extraction molybdenum antimony resistance colorimetry.

### DNA extraction, gene amplification, and MiSeq sequencing.

DNA from each soil sample (0.5 g) was extracted using QIAmp DNA minikit (Qiagen, Dusseldorf, Germany). To investigate the soil fungal composition of each sample, the ITS1 and ITS2 sequences were amplified with barcoded ITS1/ITS2 sequences (55). PCR amplification was conducted for one cycle of 94°C (1 min), 35 cycles of 94°C (30 s), 52°C (30 s), 68°C (30 s), and 6°C (10 min). High-quality samples were examined by 2% agarose gel electrophoresis and 8-cycle PCR amplification. Subsequently, PCR products were purified by gel extraction kit (AxyPrepDNA) and sequenced on the Illumina MiSeq (300-bp paired-end reads) platform (Illumina Inc., San Diego, USA).

The yielded MiSeq sequencing reads were separated based on the barcode of each sample. Any low-quality reads were filtered according to the ultrafast sequence analysis (USEARCH). The splicing sequence was qualified and filtered to optimized sequence based on the sequence parameters of maxAMBIG = 0, maxHOMOP = 8, minLength = 200, maxLength = 580 (Mothur V.1.39.5). OTU at a 97% similarity level were determined using the UPARSE pipeline (56). UNITE+INSD (UNITE and the International Nucleotide Sequence Databases) were used to identify OTU taxonomic classification. Functional annotation of specific OTU was performed using FUNGuild analysis (57).

### Data analysis.

All statistical analyses performed in the present study were conducted in the R environment (http://www.r-project.org/). ACE, Chao1, Shannon, and Simpson indexes were used to estimate the alpha diversity of soil samples according to the sequences reads from the richness and diversity of community levels using Mothur software. Beta-diversity was determined using two axes of a nonmetric multidimensional scaling (NMDS) analysis of Bray-Curtis dissimilarities based on the “vegan” package to investigate the relationships among three fungal communities. The co-occurrence network among different genera was constructed by multiple test correction (Benjamini-Hochberg, *r *> 0.75, *p-adj *< 0.01) using “igraph” and “Hmisc” packages and visualized by Gephi software. Linear discriminant analysis (LDA) was performed to determine the significant difference of soil fungal composition in pairwise comparisons (LDA > 3, *P < *0.05) using “lefser” package. Functional analysis was performed using the FUNGuild database, and significance (*P < *0.05) was compared and visualized based on “tidyverse” and “ggplot2” packages.

An analysis of variance (ANOVA) test was performed to evaluate the significance of soil properties between low and high altitudes. The relationships of soil fungal community with soil properties and plant population attributes were calculated using Spearman rank correlation analysis and presented using “pheatmap” package. In addition, to explore the effect of soil physicochemical properties on bacterial community, canonical correlation analysis (CCA) was conducted using “vegan” package. Variation in important soil properties with fungal genera was determined by linear least-squares regression analysis. The figures presented in the study were processed by Adobe Illustrator CC.

### Data availability.

The data sets can be found in online repositories: https://www.ncbi.nlm.nih.gov/bioproject/PRJNA800118.
